# Improved BM3D image denoising using SSIM-optimized Wiener filter

**DOI:** 10.1186/s13640-018-0264-z

**Published:** 2018-04-17

**Authors:** Mahmud Hasan, Mahmoud R. El-Sakka

**Affiliations:** Department of Computer Science, Western University, London, Ontario Canada

**Keywords:** Image denoising, Image restoration, BM3D, Wiener filter, Structural similarity, Collaborative filtering, Hard thresholding, Mean square error

## Abstract

Image denoising is considered a salient pre-processing step in sophisticated imaging applications. Over the decades, numerous studies have been conducted in denoising. Recently proposed *Block matching and 3D (BM3D) filtering* added a new dimension to the study of denoising. BM3D is the current state-of-the-art of denoising and is capable of achieving better denoising as compared to any other existing method. However, there is room to improve BM3D to achieve high-quality denoising. In this study, to improve BM3D, we first attempted to improve the Wiener filter (the core of BM3D) by maximizing the structural similarity (SSIM) between the true and the estimated image, instead of minimizing the mean square error (MSE) between them. Moreover, for the *DC-only BM3D* profile, we introduced a 3D zigzag thresholding. Experimental results demonstrate that regardless of the type of the image, our proposed method achieves better denoising performance than that of BM3D.

## Introduction

There are different types of noise that can contaminate a digital image. Depending on the noise type, there are various algorithms present in the literature for denoising the image. *Block matching and 3D (BM3D) filtering* [5] is one such popular algorithm that reduces *additive white Gaussian noise (AWGN)* [16] from digital images. In terms of denoising performance, BM3D is considered the best denoising filter to date. It exhibits remarkable results when compared to other existing methods. BM3D works in two identical steps. In the first step, it generates a basic estimate of the noisy image using hard thresholding. Then in the second step, it uses Wiener filter to actually denoise the noisy image. To do so, BM3D uses the basic estimate generated from the first step as an oracle (i.e., a pilot signal) in the Wiener filter.

Wiener filter is an age-old benchmark for image denoising and restoration [23]. This filter needs a degradation function for denoising or restoration. The better the degradation function is, the more denoising is achievable by Wiener filter. BM3D uses the basic estimated image from the first step as the degradation function of Wiener filter. Thus, the ultimate performance of BM3D largely depends on how good the basic estimate is.

Although BM3D achieves good denoising performance, it is not sufficient to denoise images contaminated by huge levels of noise. In other words, the performance of BM3D decreases with the increase of noise level. Again, among the different profiles of BM3D (a profile is a specific set of parameters), the *DC-only* profile (meaning that the 3D transform used is the 3D-DCT) generally performs poorer than the others. Therefore, there is scope to either propose better denoising technique than BM3D or to make BM3D perform better than what it can currently do.

The Wiener filter [23] was proposed about half a century ago. Different researchers attempted to improve the performance of the Wiener filter; however, most studies did not directly address one persisting problem of the Wiener filter which is it uses an objective function, called mean square error (MSE), which is often a misleading measure. In other words, it is possible to use a better measure than MSE as the objective function of Wiener filter. Also, if the Wiener filter can be improved, the performance of BM3D can also be improved, since it uses the Wiener filter as one of its fundamental components.

In this study, we will primarily focus on the improvement of Wiener filter. Then, we will use this improved Wiener filter in BM3D to improve its response as well. Our objective is to eventually improve the denoising performance of all profiles of BM3D through improving the Wiener filter. Note that the authors previously published their preliminary idea of how the Wiener filter can be improved [9]. In this article, the authors will utilize their previous Wiener improvement idea to further improve the BM3D filtering scheme. In addition, we will also design some additional components to improve the performance of BM3D, especially the performance of BM3D profile. It is worth mentioning that from now on till the end of the article, we will refer to *Additive White Gaussian Noise (AWGN)* whenever the term *noise* is used.

The rest of the article is organized as follows. In Section 2, we will discuss the working procedure and parameterized setup of BM3D in details. In Section 3, we will discuss the Wiener filter and its variants. In Section 4, we will address the existing problems of the Wiener filter and BM3D that we are interested to solve in this study. We will propose our methodologies in Section 5 and report their performance in Section 6. Finally, we will conclude in Section 7 discussing some possible future work.

## Block matching and 3D (BM3D) filtering

In recent years, probably the most discussed denoising technique is block matching and 3D (BM3D) filtering [5]. It was first suggested by Dabov et al. in 2007. Later, it was rigorously reviewed by Lebrun [12]. The idea has become extensively popular in denoising over the last few years. BM3D achieves excellent performance for reducing AWGN noise. In this section, we will discuss BM3D and its different profiles.

### Algorithm of BM3D

**Fig. 1:**
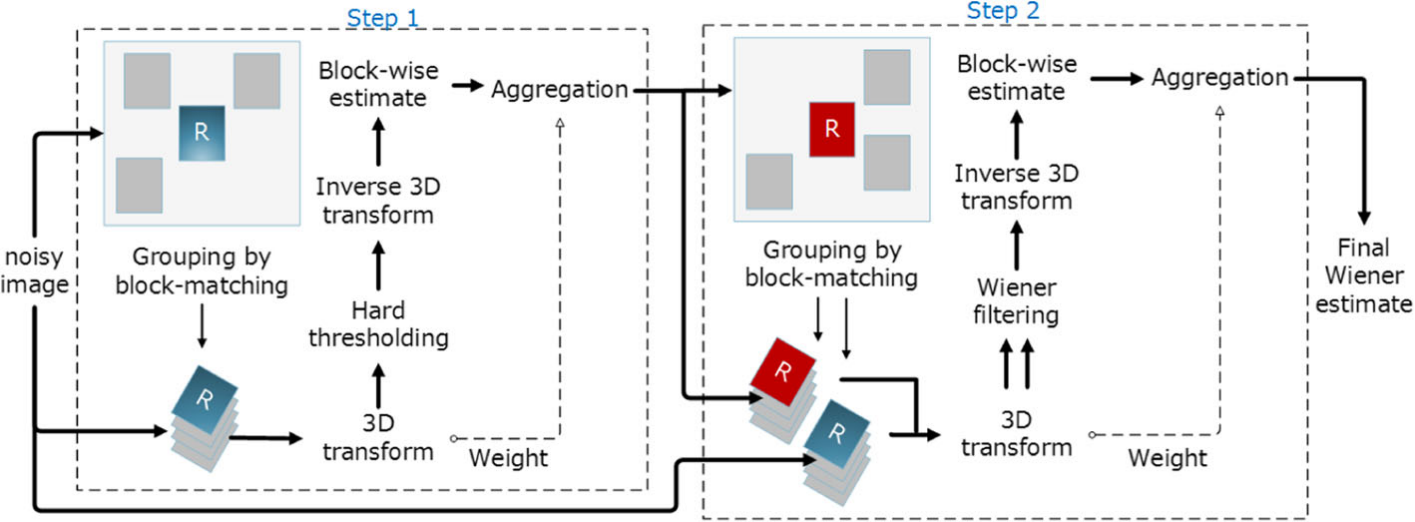
BM3D block diagram

The BM3D algorithm can be simply described in a step by step fashion. Let us start with Fig. 1 that shows the block diagram of BM3D [5].

BM3D takes the concept of non-local means (NLM) published in 2005 [2] in the sense that it also attempts denoising based on finding similar patches within a given window. BM3D has two identical steps namely step 1 and step 2. They are identical in the sense that they have no operational difference, rather the difference lies in the component that are used during the two steps. For example, the first step uses hard thresholding while the second step uses Wiener filtering. Other than that, both steps are identical. BM3D basically tries to denoise the noisy image in the first step to generate a basic estimate. This basic estimate is used in Wiener filtering of the second step as an oracle (i.e., degradation model) [5].

#### BM3D first step

In step 1, BM3D starts its operation by dividing the noisy image into a number of blocks or patches. For each patch, it then generates a window centering the block being processed. BM3D defines this center patch as a reference patch. Then, within this window, BM3D looks for the patches similar to the reference patch. Usually, a good number of similar patches are found. BM3D defines a threshold that decides whether two patches are similar or not (see Table 1). Once the similar patches are found, BM3D stacks all the similar patches together thus building a 3D block, where the first patch is the reference patch and others patches are sorted according to their distance to the reference patch. BM3D allows a number of 3D transform techniques to transform the domain from spatial to frequency (as indicated by the 3D transform in Fig. 1). After the 3D transform is performed, the most important part of the first step, known as hard thresholding, is executed. Hard thresholding is a filter that allows any coefficient with absolute value above a certain threshold to pass through, while converts the remaining coefficients to zero. This is the only operation in the first step that performs denoising, the rest are only to make a platform for hard thresholding. After hard thresholding, BM3D tries to generate a basic estimate. For this, it needs to transform the block coefficients to intensity values in the spatial domain. This is known as inverse 3D transform. After performing the inverse 3D transform, what is obtained is the 3D block that we started working with. But this time, it is denoised. Now, for each patch in the 3D block, an aggregation to estimate the reference patch is made. This aggregation is simply taking different weights and estimating each pixel. Once the aggregation is done, the basic estimate is ready to start the second step.

**Table 1:** Parameterized setup for the wavelet profile of BM3D

			Fast profile	Normal profile	
	Notations	Meaning		*σ*≤40	*σ*>40
Parameters for	$\tau ^{ht}_{2D}$	2D transform used	2D-Bior1.5	2D-Bior1.5	2D-DCT
step 1 (ht)	$N^{ht}_{1}$	Patch size	8	8	12
	$N^{ht}_{2}$	Maximum number of	16	16	16
		similar patches retained			
	$N^{ht}_{step}$	Step of ref. patch	6	3	4
	$N^{ht}_{S}$	Size of search window	25	39	39
	$N^{ht}_{FS}$	Exhaustive search window size	6	1	1
	$N^{ht}_{PR}$	Predictive search window size	3	-	-
	*β* ^*ht*^	Parameters for Kaiser window	2.0	2.0	2.0
	*λ*_2*D*_	Pre-processing threshold	0	0	2.0
	*λ*_3*D*_	Hard threshold	2.7	2.7	2.8
	$\tau ^{ht}_{match}$	Similarity threshold for patches	2500	2500	5000
Parameters for	$\tau ^{wie}_{2D}$	2D transform used	2D-DCT	2D-DCT	2D-DCT
step 2 (wie)	$N^{wie}_{1}$	Patch size	8	8	11
	$N^{wie}_{2}$	Maximum number of	16	32	32
		similar patches retained			
	$N^{wie}_{step}$	Step of ref. patch	5	3	6
	$N^{wie}_{S}$	Size of search window	25	39	39
	$N^{wie}_{FS}$	Exhaustive search window size	5	1	1
	$N^{wie}_{PR}$	Predictive search window size	2	-	-
	$\tau ^{wie}_{match}$	Similarity threshold for patches	400	400	3500
	*β* ^*wie*^	Parameters for Kaiser window	2.0	2.0	2.0
Common			1D-Haar	1D-Haar	1D-Haar

In theory, it is obvious that the more patches are present in the 3D block, the better estimates will be found for one single pixel as well as the better denoised basic estimates. However, according to Dabov et al. [5], in practice, it is seen that after a certain number of similar patches, BM3D does not seem to perform better.

#### BM3D second step

The second step is similar to the first step with two small differences. First, the 3D grouping is now performed on a basic estimate that was obtained from the first step, not on the noisy image as in step 1. Second, the hard thresholding is not used any more after the 3D transform. Instead, a Wiener filter is now used. We will discuss in Section 3 that the Wiener filter needs a degradation function *H*() to work. In BM3D, the 3D group built on the basic estimate is considered as the degradation function for BM3D while the corresponding 3D group of the noisy image is the degraded image function *G*().

Equation 1 shows how the Wiener filter works in BM3D. Here, $\mathbb {P}^{basic}(P)(\xi)$ is the 3D block from the basic image and $\mathbb {P}(P)$ is the corresponding 3D block from the noisy image. $\tau ^{wien}_{3D}$ denotes the 3D transformation for the Wiener filter phase. Once the inverse 3D transform of Eq. 1 is computed, $\mathbb {P}^{wien}(P)$ is found which is the final estimate for one block. Once the estimate is obtained, a weighted aggregation operation, like in step 1, is performed to build the final denoised image.

1 $$ \omega_{p}(\xi)=\frac{\left | \tau^{wien}_{3D}\mathbb{P}^{basic}(P)(\xi) \right |^{2}}{\left | \tau^{wien}_{3D}\mathbb{P}^{basic}(P)(\xi) \right |^{2}+\sigma^{2}}\cdot \tau^{wien}_{3D}\mathbb{P}(P)  $$

#### Parameters and profiles of BM3D

The performance of BM3D varies depending on the type of transformation used in both steps. There are two major profiles of BM3D, namely: *DC-only profile* and *wavelet profile*. In DC-Only profile, BM3D uses a three-dimensional discrete cosine transform as a 3D transform. For the wavelet profile, on the other hand, BM3D uses a combination of 2D bi-orthogonal transform and 1D-Haar or Walsh-Hadamard transform. DC-only profile generally produces poorer results as compared to its wavelet counterpart [5, 6]. Wavelet profile may be defined as the *mainstream BM3D* since the authors of BM3D [5] recommended to use Wavelet transform in their proposed denoising method. This is because, the main stream BM3D has PSNR gain much better than DC-only profile. For the wavelet profile, we used an exactly same parameterized setup as in BM3D [5]. We present the basic parameterized setup from the original article [5] in Table 1 for readers’ convenience. We use exactly the same parameters to ensure the same environment for the experiment. The wavelet profile uses two sub-profiles called *normal profile* and *fast profile*. The only difference between them is that the denoising performance is compromised in order to reduce the computational complexity in *fast profile*. Another difference between these two profiles is the *fast profile* uses predictive searching in order to decrease its searching time while the *normal profile* uses only exhaustive searching.

## Wiener filter revisited

A Wiener filter provides an opportunity to deal with both noises and degradation. This feature makes the Wiener filter unique in both image denoising and restoration. This filter is also called *minimum mean square error*. This is because the core idea behind the Wiener filter is to satisfy an objective function which is the mean square error (MSE). In other words, this guarantees that the image restored by the Wiener filter $\hat {f}$ will have minimum MSE with respect to original uncorrupted image *f*. Equation 2 shows that the expectation of a Wiener filter is to have minimum MSE between *f* and $\hat {f}$.

2 $$ e^{2}=E{(f-\hat{f})^{2}}  $$

The Wiener filter is defined by Eq. 3. Note that all terms are given in the transformed domain. Here, *H*(*u,v*) is the degradation function, *H*^∗^(*u,v*) is the conjugate complex of *H*(*u,v*), *S*_*n*_ is the power spectrum of noise defined as $S_{n}=\left |{N(u,v)} \right |^{2}$, and *S*_*f*_ is the power spectrum of undegraded image defined as $S_{f}=\left |{F(u,v)} \right |^{2}$. *G*(*u,v*) is the transform of the degraded image, and $\hat {F}(u,v)$ is the final estimate for the restored image. Once the inverse transform of $\hat {F}(u,v)$ is computed, $\hat {f}(x,y)$ is obtained which is the denoised/restored approximation for original image *f*(*x,y*).

3 $$ \hat{F}(u,v)=\frac{H^{*}(u,v)}{\left |H(u,v) \right |^{2}+\frac{S_{n}}{S_{f}}}G(u,v)  $$

Comparing Eq. 1 with Eq. 3, it is evident that both equations are exactly the same, except that Eq. 1 works with 3D data. Equation 3 can be solved for *G*(*u,v*) as in Eq. 4 and rewrite Eq. 4 as in Eq. 5.

4 $$ G(u,v)=\frac{H^{*}(u,v)S_{f}(u,v))}{\left | H(u,v)\right |^{2}S_{f}(u,v)+S_{n}(u,v)}  $$

5 $$ G(u,v)=\frac{1}{H(u,v)}\left[ \frac{\left | H(u,v) \right |^{2}}{\left | H(u,v) \right |^{2}+\frac{S_{n}(u,v)}{S_{f}(u,v)}} \right]  $$

Now, if the noise is zero, the term inside the square brackets in Eq. 5 becomes 1, which means the Wiener filter is reduced to an inverse filter and works for only restoration. However, if there is noise, the Wiener filter incorporates itself for removal of noise along with restoration. This is what makes the Wiener filter unique.

## Existing problems with Wiener filter and BM3D

### Wiener filter objective function

#### MSE-optimized Wiener filter

As stated in Section 3, a Wiener filter tries to minimize its objective function shown in Eq. 2 while denoising/restoring a degraded image. This function is also known as mean square error (MSE) as defined in Eq. 6.

6 $$  MSE=\frac{1}{m\times n}\sum\limits_{i=0}^{m-1}\sum\limits_{j=0}^{n-1}[I(i,j)-\hat{I}(i,j)]^{2}  $$

In this Equation, *I* and $\hat I$ are considered as the true (or noise free) image and the reconstructed (or denoised) image, respectively. As the difference between these two gets smaller the closer the images are. Also, increased closeness indicates a more accurately denoised image. With this fundamental property, MSE is being used as an image quality metric [13].

The Wiener filter (Eq. 3) guarantees that the denoised/restored image is the closest image possible to the true undegraded image, since this filter is optimized for MSE. We might conclude at this point that in the best-case scenario the Wiener filter may reconstruct an image whose MSE with respect to the true image is zero. That is, both images are exactly the same. However, there are still problems with the typical use of MSE that prevent the Wiener filter from achieving more accurate and perfect results.

#### Structural similarity

**Fig. 2:**
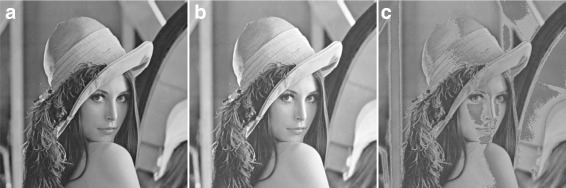
The MSE effect on brightness increase: **a** Original Lena image. **b** Lena image after adding a constant value of 30 to all pixel values to increase the brightness. **c** Lena image after subtracting a constant value of 30 from any pixel value less than 150 and adding a constant value of 30 otherwise; the MSE between the images in **a** and **b** is equal to 900, while the MSE between the images in **a** and **c** is also 900

Wang et al. [20] showed that although MSE is a good quality measure, it is sometimes seriously misleading. This is because MSE does not consider anything other than the point-to-point distance [21]. For example, if we add a constant value to all the pixel values of an image just to increase its brightness, the images are *visually* exactly the same; however, MSE still generates a huge error because of point-to-point distance measurement. Figure 2 shows such an example of misleading of MSE measure. In this figure, we used the well-known Lena image in Fig. 2a and added a constant 30 to all of its pixel values in order to increase its brightness. The brightened image is shown in Fig. 2b. Although there is no visual distortion in the image, the calculated MSE between them is 900. This value is not negligible, and hence MSE cannot be considered as a true error measure.

Now, let us also consider Fig. 2c. This is similar to Fig. 2b except that now we have not just added a positive constant to all brightness levels, instead, if the brightness value is less than 150, we subtracted 30 from it; otherwise, we added 30. Due to the presence of the square term in Eq. 6, the MSE still generates the same error value (900) as in Fig. 2, even though the image in Fig. 2c is greatly distorted, if it is compared with the image in Fig. 2b.

To avoid such misleading error situations, Wang et al. [20] proposed that modern image quality measurement metrics should not depend only on point-to-point distances, it should also consider the geometric or structural similarity between the images. Otherwise, there is a possibility to have the same error results for different images as depicted above. Thus, they proposed a new error measurement metric called *structural similarity (SSIM)*. Their proposed error measure, SSIM, is defined in Eq. 7.

7 $$  SSIM(x,y)=\frac{(2\mu_{x}\mu_{y}+c_{1})(2\sigma_{xy}+c_{2})}{\left(\mu_{x}^{2}+\mu_{y}^{2}+c_{1}\right)\left(\sigma_{x}^{2}+\sigma_{y}^{2}+c_{2}\right)}  $$

In this Equation, *x* and *y* denote two blocks of the same position from the true image and the reconstructed image. *μ*_*x*_ and *μ*_*y*_ denote the arithmetic mean or average of *x* and *y* blocks, respectively. $\sigma _{x}^{2}$ and $\sigma _{y}^{2}$ indicate the variance of *x* and *y* blocks, respectively, while *σ*_*x*_*y* is the co-variance between *x* and *y*. *c*_1_ and *c*_2_ are two constants to stabilize the division with weak denominator and their values are calculated using Eqs. 8 and 9, respectively. Here, *L* is the dynamic range of the image while *k*_1_ and *k*_2_ are two constants whose values are *k*_1_=0.01 and *k*_2_=0.03, respectively [20, 21].

8 $$  c_{1}={(k_{1}L)}^{2}  $$

9 $$  c_{2}={(k_{2}L)}^{2}  $$

This measure is calculated block by block. Therefore, a mean SSIM of all blocks is used to represent the final SSIM index value for the whole image. The SSIM index generally varies between − 1 to + 1 which is often taken as absolute to avoid a negativity value. Thus, the SSIM index is a fractional number between 0 and 1 (inclusive), where a 0 indicates *no similarity* and a 1 indicates *exact similarity* between two images. Using this measure, we have 0.9650 and 0.7850 for Fig. 2b, c, respectively, which indicates that Fig. 2c is much more distorted than Fig. 2b. This distortion was overlooked by MSE.

There are other misleading measures of MSE; however, we will not cover them in this study. For a detail list of misleading characteristics of MSE, we refer the reader to the literature [10, 20–22].

### Existing problems of BM3D

#### Higher noise levels

BM3D has poor denoising performance to images corrupted by higher levels of noise as compared to its response to images corrupted by lower levels of noise. In other words, BM3D’s performance decreases as the noise level increases.

#### Poor performance of BM3D for DC-only profile

BM3D requires the hard thresholding to be performed in a transform domain, after the 3D transform is performed on the similar image blocks. This 3D transform can be either a 2D+1D transform or a 3D transform. In BM3D, usually a 3D Wavelet is used as 3D transform or a combination of 2D-DCT plus a 1D wavelet transform. However, the transform choice is not restricted. If the 3D-DCT is used, this profile is called DC-only profile. Note that, a profile is basically a set of parameters that is used in BM3D. In the DC-only profile, coefficients are categorized into two categories: DC and AC coefficients, where the DC coefficient preserves the average of the block intensity, which is a significant piece of block information. It is worth mentioning that the DC coefficient might also possess some noise. When BM3D uses hard thresholding to get rid of the noise in the transform domain, it does not really treat the AC and the DC coefficients differently. As a result of this, the final outcome of DC-only profile is poorer than other profiles (e.g., wavelet profiles).

## Proposed method

### SSIM-optimized Wiener filter

From the discussion in Section 4.1, it is evident that the MSE is not adequate for assessing the closeness between two images, it is rather good at assessing the distance between them. Instead, the SSIM is a more acceptable alternative. This is because, MSE deals with image data while SSIM deals with image information.

It should be noted that the idea of optimizing the Wiener filter with other quality measurement objective functions were tested by a number of objective functions such as sum of absolute difference (SAD) and median of absolute difference (MAD). However, since most of the similarity measures are rather *closeness* measures based on differences of pixels’ intensities and not on visual similarities, they could not actually further optimize the Wiener filter. Therefore, the choosing of SSIM as an objective function was logical.

The optimization of a denoising filter by SSIM instead of MSE is not very recent. Channappayya et al. in [3, 4] showed that any linear filter can be optimized by SSIM. They compared their proposed SSIM-optimized filter’s result with the MSE-optimized Wiener filter. Their reported results showed that they were able to achieve a higher SSIM value compared to the MSE-optimized Wiener filter; however, their PSNR gain was still poorer than the MSE-optimized Wiener filter.

An SSIM-optimized Wiener filter, which we proposed earlier [9], considers the structural similarity between the reconstructed image and the true image. Since higher SSIM index indicates more similar images, our proposed Wiener filter’s target is to estimate an image which has maximum SSIM possible. In this case, the expectation function of the Wiener filter becomes Eq. 10 which now needs to be maximized. In Eq. 10, *E* is the expectation and *f* and $\hat {f}$ are the true and reconstructed images, respectively.

10 $$  e=E\{ssim(f,\hat{f})\}  $$

Having defined the expectation function, our target is to ensure that our designed Wiener filter maximizes our expectation. Taking a careful look at Eq. 3, we realize that replacing the term $\frac {S_{n}}{S_{f}}$ by a variable *K* is reasonable and finding a suitable value of *K* is possible [7]. Therefore, for our proposed case, we can start with the lowest possible value of *K* and loop through the highest possible value of *K*. For each *K*, we record the SSIM error measure in a vector and then restore the image using that *K* for which the error has been recorded maximum. Thus, for a given range of noise level, it is guaranteed that our proposed Wiener filter should be SSIM optimized. Likewise, it should also provide better denoising and restoration.

Since the core of our proposed improvement over BM3D is the SSIM-optimized Wiener filter, interested readers may want to compare the performance given by both SSIM- and MSE-optimized Wiener filter. We refer the reader to our previously published work [9].

### 3D zigzag thresholding

In the discrete cosine transform, the first coefficient is basically the average of all pixel values within a given block [17]. Therefore, for a precise inverse transforming result, the accurate DC coefficient is crucial. In order to make sure that the inverse transformation result is the product of vital block information, our proposed method does not apply hard thresholding on the DC coefficient. Instead, it only applies hard thresholding on AC coefficients. The AC coefficients carry various block frequency information [1, 7, 17]. This information varies from low-frequency to high-frequency information. Thresholding all AC coefficients in the same manner might lead to losing some significant information, while preserving some other insignificant information.

In order to keep the most meaningful block information and just reduce the noise, we should use a zigzag thresholding, instead of a hard thresholding. Zigzag thresholding is realized by applying little or no thresholding on the DC coefficient and first few AC coefficients and then applying an increasingly higher thresholding on the rest of the AC coefficients (the higher the frequency, the more thresholding is applied). Determining the actual zigzag thresholding value can be chosen using a gamma curve. A gamma curve is as simple as *λ*_3*D*_=*κ*^*γ*^, where *κ* is the coefficient value, *γ* is a positive value ≥ 1 that is directly proportional to the coefficient number, and *λ*_3*D*_ is the thresholded value. When *γ*=1, the relation becomes linear. Using this thresholding scheme, we gradually increase the thresholding effect with the increase of the coefficient number. Note that this *3D zigzag thresholding* proposal applies to only DC-only profile of BM3D and not to the actual wavelet profile.

## Results and discussion

### Data set and parameterized setup

**Fig. 3:**
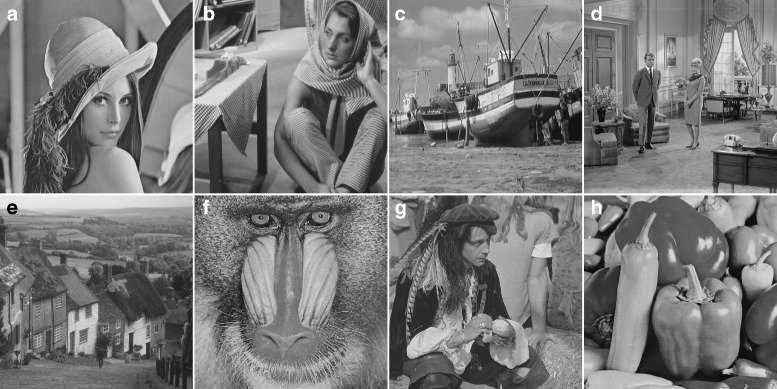
Test image set: **a** Lena, **b** Barbara, **c** Boat, **d** Living room, **e** Goldhill, **f** Baboon, **g** Pirate, and **h** Peppers

We used eight standard gray scale test images for our experiment. The images used are shown in Fig. 3.

For all these images, we recorded the responses of a MSE-optimized Wiener filter and our proposed SSIM-optimized Wiener filter. All performance comparison tables reported in this article are based on the average performance on these eight test images for each noise level. The noise level is generated using Eq. 11 where the value of *σ* is varied from 0 (minimum) to 100 (maximum) with *μ*=0 (zero-mean). Here, *σ* is known as noise standard deviation. All other parameters are used with their default values as suggested by Dabov et al. [5] and presented in Table 1.

11 $$  p(z)=\frac{1}{\sqrt{2\pi \sigma }}e^{-\frac{(x-\mu)^{2}}{2\sigma^{2}}}  $$

Experimental results presented in this article are reported in both subjective and objective forms. We used peak signal to noise ratio (PSNR) and structural similarity (SSIM) as our objective measure. As for the subjective assessment, the output from BM3D and from our proposed method is used to visually comapre the performance. While we present the result of all noise level experiments in the objective measure, we only present one noise level in the subjective assessment. We did so in order to keep the number of pages within the allowable limit.

### Performance analysis of wavelet profile

#### Normal profile

**Fig. 4:**
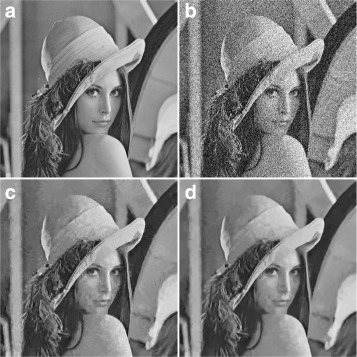
Subjective assessment between the normal profile of BM3D and the proposed method. **a** Original image. **b** Noisy image at noise level *σ*=50, *PSNR*=14.60 and *SSIM*=0.1163. **c** Output using the normal profile of BM3D, *PSNR*=28.29 and *SSIM*=0.7455. **d** Output using the proposed method, *PSNR*=28.97 and *SSIM*=0.7972

Since the Wavelet profile itself exploits higher performance as compared to the DC-only profile, even a small increase in PSNR/SSIM indicates a reasonable improvement. The experimental results for this profile are given in Table 2. The subjective measure is given in Fig. 4 for Lena image with *σ*=50.

**Table 2:** Performance comparison of normal profile and proposed method

Noise	BM3D	Proposed	PSNR	% PSNR	BM3D	Proposed	SSIM	% SSIM
level	PSNR	PSNR	gain	gain	SSIM	SSIM	gain	gain
10	34.17	34.16	– 0.01	– 0.029%	0.903	0.903	0.000	0.000%
20	31.04	31.10	0.06	0.002%	0.843	0.844	0.001	0.119%
30	29.08	29.28	0.20	0.688%	0.789	0.795	0.006	0.760%
40	27.42	27.87	0.45	1.641%	0.731	0.751	0.020	2.736%
50	26.79	27.05	0.26	0.971%	0.702	0.719	0.017	2.422%
60	25.85	26.28	0.43	1.663%	0.656	0.690	0.034	5.183%
70	25.06	25.65	0.59	2.354%	0.615	0.664	0.049	7.967%
80	24.37	25.11	0.74	3.037%	0.575	0.641	0.066	11.478%
90	23.70	24.59	0.89	3.755%	0.534	0.617	0.083	15.543%
100	23.15	24.18	1.03	4.449%	0.500	0.600	0.100	20.000%

#### Fast profile

**Fig. 5:**
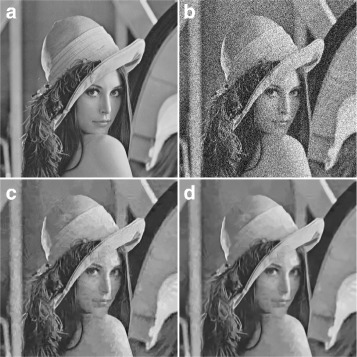
Subjective assessment between the fast profile of BM3D and the proposed method. **a** Original image. **b** Noisy image at noise level *σ*=50, *PSNR*=14.60, and *SSIM*=0.1163. **c** Output using the normal profile of BM3D, *PSNR*=28.28 and *SSIM*=0.7438. **d** Output using the proposed method, *PSNR*=28.93 and *SSIM*=0.7967

The fast profile is similar to the normal profile, except that this profile is faster, as the identification of similar blocks is predicted. The performance of the fast profile is slightly lower than normal profile. The experimental results obtained for fast profile is presented in Table 3. Figure 5 shows a subjective measure for the Lena image with *σ*=50.

**Table 3:** Performance comparison of fast profile and the proposed method

Noise	BM3D	Proposed	PSNR	% PSNR	BM3D	Proposed	SSIM	% SSIM
level	PSNR	PSNR	gain	gain	SSIM	SSIM	gain	gain
10	34.18	34.17	–0.01	–0.029%	0.904	0.903	–0.001	–0.111%
20	31.04	31.09	0.05	0.161%	0.844	0.844	0.000	0.000%
30	29.07	29.27	0.20	0.688%	0.789	0.795	0.006	0.760%
40	27.45	27.91	0.46	1.676%	0.732	0.752	0.020	2.732%
50	26.81	27.06	0.25	0.932%	0.703	0.720	0.017	2.418%
60	25.89	26.30	0.41	1.583%	0.658	0.690	0.032	4.863%
70	25.07	25.63	0.56	2.234%	0.614	0.663	0.049	7.980%
80	24.38	25.11	0.73	2.994%	0.575	0.641	0.066	11.478%
90	23.76	24.64	0.88	3.704%	0.534	0.621	0.087	16.292%
100	23.14	24.17	1.03	4.451%	0.500	0.600	0.100	20.000%

### Extension of wavelet profile for color image denoising

One approach to employ *Color BM3D (CBM3D)* to 24-bit true color images is to apply BM3D separately on each of its channels. However, correct grouping is one of the key properties of BM3D and it largely depends on the noise level. Again, grouping is a time consuming operation, doing it thrice makes the algorithm considerably slower. Moreover, three channels should generate three different groupings with sparsity of image information which lead to erroneous hard thresholding. BM3D extension to color image denoising is realized by converting a noisy RGB image into a luminance and chrominance transformed space. In this transformed space, the luminance signal contains most of the image information while the chrominance signals contain low-frequency information. Therefore, CBM3D performs grouping on luminance channel only and uses exactly the same grouping for chrominance channels. The idea behind this form of grouping is that if the luminance of two blocks are mutually similar, then the chrominance of these blocks are also mutually similar [5].

**Fig. 6:**
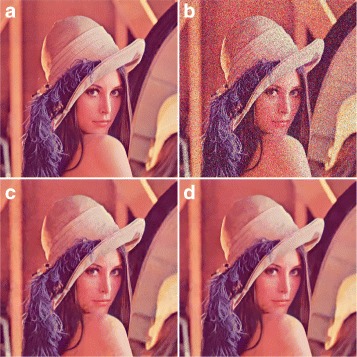
Subjective assessment between the color normal profile of BM3D and the proposed method. **a** Original image. **b** Noisy image at noise level *σ*=50, *PSNR*=14.81, and *SSIM*=0.5495. **c** Output using the normal profile of BM3D, *PSNR*=29.55 and *SSIM*=0.9741. **d** Output using the proposed method, *PSNR*=29.89 and *SSIM*=0.9759

We used the same concept for color image denoising as in CBM3D except that in step 2, we used our improved Wiener filter instead of the existing Wiener filter. The experiments are performed for both normal and fast profiles. The experimental results for normal color profile are presented in Table 4. For visual inspection of denoised color images of normal profile of Lena image with *σ*=50, we refer the reader to Fig. 6.

**Table 4:** Performance comparison of color profile (normal) and proposed method

Noise	BM3D	Proposed	PSNR	% PSNR	BM3D	Proposed	SSIM	% SSIM
level	PSNR	PSNR	gain	gain	SSIM	SSIM	gain	gain
10	34.11	34.11	0.00	0.000%	0.937	0.937	0.000	0.000%
20	31.29	31.35	0.06	0.192%	0.896	0.897	0.001	0.112%
30	29.56	29.70	0.14	0.474%	0.860	0.864	0.004	0.465%
40	27.92	28.14	0.22	0.788%	0.818	0.824	0.006	0.733%
50	27.61	27.80	0.19	0.688%	0.798	0.806	0.008	1.003%
60	26.81	27.06	0.25	0.932%	0.768	0.781	0.013	1.693%
70	26.07	26.43	0.36	1.381%	0.737	0.757	0.020	2.714%
80	25.47	25.93	0.46	1.806%	0.710	0.737	0.027	3.803%
90	24.89	25.42	0.53	2.129%	0.682	0.717	0.035	5.132%
100	24.23	24.85	0.62	2.558%	0.649	0.692	0.043	6.626%

**Fig. 7:**
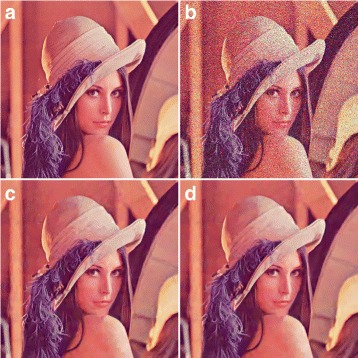
Subjective assessment between the color fast profile of BM3D and the proposed method. **a** Original image. **b** Noisy image at noise level *σ*=50, *PSNR*=14.81, and *SSIM*=0.5495. **c** Output using the fast profile of BM3D, *PSNR*=29.29 and *SSIM*=0.9724. **d** Output using the proposed method, *PSNR*=29.57 and *SSIM*=0.9740

For the fast color profile, the experimental results are shown in Table 5. The output is shown in Fig. 7 for Lena image with *σ*=50 for a visual inspection of the reader. Since the fast profile compromises the performance in order to reduce time, the output is poorer than the normal profile. Therefore, for the image presented for *σ*=50 in Fig. 7, it may not depict a visible difference; however, this will be evident at higher noise levels.

**Table 5:** Performance comparison of color profile (fast) and proposed method

Noise	BM3D	Proposed	PSNR	% PSNR	BM3D	Proposed	SSIM	% SSIM
level	PSNR	PSNR	gain	gain	SSIM	SSIM	gain	gain
10	33.95	33.96	0.01	0.029%	0.936	0.936	0.000	0.000%
20	31.00	31.09	0.09	0.290%	0.893	0.894	0.001	0.112%
30	29.05	29.28	0.23	0.792%	0.852	0.857	0.005	0.589%
40	27.32	27.59	0.27	0.988%	0.804	0.811	0.007	0.871%
50	27.38	27.54	0.16	0.584%	0.795	0.802	0.007	0.881%
60	26.50	26.77	0.27	1.019%	0.765	0.775	0.010	1.307%
70	25.78	26.12	0.34	1.319%	0.738	0.752	0.014	0.543%
80	25.13	25.57	0.44	1.751%	0.710	0.730	0.020	2.817%
90	24.42	24.96	0.54	2.211%	0.681	0.707	0.026	3.818%
100	23.69	24.22	0.53	2.237%	0.648	0.676	0.028	4.321%

### Performance analysis of DC-only profile

**Fig. 8:**
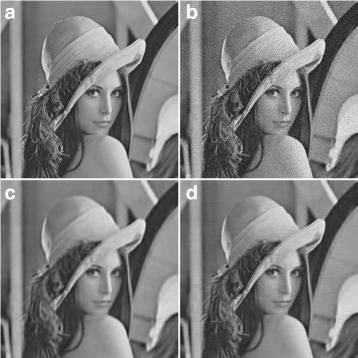
Subjective assessment between the DC-only profile of BM3D and the proposed method. **a** Original image. **b** Noisy image at noise level *σ*=20, *PSNR*=22.13, and *SSIM*=0.3402. **c** Output using the DC-only of BM3D, *PSNR*=28.21 and *SSIM*=0.7883. **d** Proposed Method’s Output, *PSNR*=29.65 and *SSIM*=0.8155

Figure 8 shows a comparison between the performance of the proposed zigzag thresholded result and that of BM3D for DC-only profile, where Fig. 8a is the original image, Fig. 8b is the noisy image (*σ*=20), and Fig. 8c, d are the output produced by BM3D (DC-only) and the proposed method, respectively. The results show an improvement in the quality of the denoised image when the proposed method is applied, especially at the face and shoulder areas. For further experimental results on the performance of DC-only profile, readers are referred to Hasan [8].

### Discussion

So far, we have presented a portion of our objective and subjective results from our experimentation. We have also compared our proposed method with the original BM3D. Objectively, our proposed method was able to produce higher PSNR and SSIM values than that of the original BM3D method. Tables 2, 3, 4, and 5 show that as the amount of noise increases, the proposed method managed to produce better denoising output, when compared to the original BM3D. For monochrome images, the *SSIM gain* by the proposed method was as high as 20% while the *PSNR gain* was as high as 4.49*%*. For color images, we achieved slightly less, as the maximum SSIM and PSNR gain were 6.63 and 2.56*%*, respectively. Subjectively, the quality of the denoising by the proposed method was better than that by the original BM3D. This was especially visible in the flat areas of the images, e.g., Lena’s shoulder and face. It is evident from the results that our proposed method achieved better objective and subjective denoising quality.

## Conclusions

In this study, we rigorously reviewed the current state-of-the-art image denoising scheme (BM3D) as well as its core component, the *Wiener filter*. We proposed an improved Wiener filter optimized for SSIM that has essentially improved the performance of BM3D. Through a number of experiments, we proved that our proposed Wiener filter, when used in BM3D, is capable of achieving high-quality image denoising. Our idea works for both monochrome (gray scale) and color images. As a brief summary, our novel contributions in this study are the following: (1) reviewing the state-of-the-art image denoising method BM3D with its components and profiles, (2) finding its existing shortcomings, (3) suggesting an improved Wiener filter optimized for SSIM [9], (4) using the SSIM-optimized Wiener in BM3D as its core component, and (5) thereby proving that the performance of original BM3D has been significantly improved in our proposed method (through detailed comparative studies via calculating a number of performance measurement metrics). (6) In addition, we have also proposed a technique named 3D zigzag thresholding for improving the poor performance of DC-only profile of BM3D. All the innovations are discussed in detail in Section 5 while the performed tests, measurement metrics, and obtained results are discussed in Section 6.

### Future direction

There are other variants of recently proposed SSIM called multi-scale structural similarity (MS-SSIM) [19]. Also, there are other image quality measurement metrics such as image quality index (IQI) [18], Normalized Correlation [11], Sum of Absolute Differences, and many others [15]. A possible future work will test our proposed method by using all of these measures. Recently, BM3D has been extended for video denoising. Also there is BM4D [14]. Since our idea is to change the core of *BMxD* in general, a study of assessing our method in all *BMxD* versions will be considered. In addition, there are recent works such as finding visually similar images using a convolutional deep neural network [24], it would be interesting to study if finding a similarity between images with deep neural networks helps in forming a quality metric that can eventually be used in a Wiener filter. Also, applying our improved denoising method could help in reducing noise for applications such as text extraction from complex background images [25].

## Abbreviations

AWGN: Additive white Gaussian noise
BM3D: Block matching and 3D filtering
BM4D: Block matching and 4D filtering
BMxD: Block matching and xD filtering
CBM3D: Color block matching and 3D filtering
DCT: Discrete cosine transform
IQI: Image quality index
MAD: Median of absolute difference
MS-SSIM: Multi-scale structural similarity
MSE: Mean square error
NLM: Non-local means
PSNR: Peak signal to noise ratio
RGB: Red-green-blue
SAD: Sum of absolute difference
SSIM: Structural similarity
